# Supplementary material for: The Diverse Potential of Gluten from Different Durum Wheat Varieties in Triggering Celiac Disease: A Multilevel In Vitro, Ex Vivo and In Vivo Approach
Source: Nutrients. 2020 Nov 20;12(11):3566. doi: 10.3390/nu12113566 (PMC7699868; doi:10.3390/nu12113566)
Supplement: Supplementary file 1 [file nutrients-12-03566-s001.pdf]

Supplementary Material

Table S1. Calculated statistical indices of identified peptides triggering celiac disease.

	N	Mean	Std. Deviation	Minimum	Maximum	Range	Kurtosis	Skewness	Variance	Significance
**IP1**	82	120.90	26.34	74.25	177.95	103.71	-0.65	0.09	693.65	***
**IP2**	82	122.66	142.82	39.07	1 372.56	1 333.49	74.89	8.47	20 396.61	***
**IP3**	82	43.40	13.10	20.29	72.05	51.76	- 0.90	0.06	171.66	***
**IP4**	82	339.10	106.62	133.67	554.16	420.49	-0.93	0.02	11 368.01	***
**IP5**	82	189.77	74.42	56.25	366.27	310.02	- 0.68	0.14	5 538.47	***
**IP6**	82	35.72	18.89	3.91	100.12	96.20	1.16	0.85	356.99	***
**IP7**	82	57.67	27.41	8.65	157.08	148.43	1.29	0.89	751.11	***
**TP1**	82	15.06	5.54	5.29	34.64	29.35	0.87	0.59	30.69	***
**TP2**	82	45.76	20.52	9.49	127.76	118.27	1.79	0.86	421.12	***
**TP3**	82	5.33	5.16	0.65	31.23	30.58	9.66	2.75	26.63	***
**TIP**	82	909.21	335.18	360.43	2 648.85	2 288.42	7.60	1.67	112 346.96	***
**TTP**	82	66.15	28.91	16.62	193.63	177.01	3.29	1.14	835.50	***
**TIT**	82	975.36	354.30	377.05	2 722.60	2 345.55	5.96	1.43	125 529.40	***

Results are expressed in ppm (μg of peptide per gram of sample). Peptides, which contain sequences able to trigger the adaptive immune response, are indicated with the code "IP", while peptides, which contain sequences able to trigger the innate immune response, are indicated with the code "TP". IP1: TQQPQQPFPQ; IP2: SQQPQQPFPQPQ; IP3: QAFPQQPQQPFPQ; IP4: TQQPQQPFPQQPQQPFPQ; IP5: PQTQQPQQPFPQFQQPQQPFPQPQQP. IP1: TQQPQQPFPQ; IP2: SQQPQQPFPQPQ; IP3: QAFPQQPQQPFPQ; IP4: TQQPQQPFPQQPQQPFPQ; IP5: PQTQQPQQPFPQFQQPQQPFPQPQQP. IP6: FPQQPQLPFPQQPQQPFPQPQQPQ; IP7: QQPQQPFPQPQQTFPQQPQLPFPQQPQQPF; TP1: LQPQNPSQQQPQ; TP2: RPQQPYPQPQPQ. TP3: LQPQNPSQQQPQEQVPL. TIP, sum of peptides, which contain sequences able to trigger adaptive immune response; TTP, sum of peptides, which contain sequences able to trigger innate immune response; TIT, sum of peptides, which contain sequences able to trigger immune response in celiac people. Significance correspond to the result of one-way ANOVA ***: $p \leq 0.001$
